# Prognostic impact of multidrug resistance gene expression on the management of breast cancer in the context of adjuvant therapy based on a series of 171 patients

**DOI:** 10.1038/sj.bjc.6602958

**Published:** 2006-01-24

**Authors:** L Moureau-Zabotto, S Ricci, J P Lefranc, F Coulet, C Genestie, M Antoine, S Uzan, J P Lotz, E Touboul, R Lacave

**Affiliations:** 1 Service d'Oncologie Radiothérapie, Hôpital Tenon, AP-HP, Cancerest, GHU EST, Université Paris VI, 4 rue de la Chine, Paris 75020, France; 2 Service d'Histologie-Biologie tumorale, Hôpital Tenon, AP-HP, Cancerest, GHU EST, Université Paris VI, 4 rue de la Chine, Paris 75020, France; 3 Service de Chirurgie Mammaire et Gynécologique, Groupe Hospitalier Pitié Salpêtrière, AP-HP, GHU EST, 47-83 Boulevard de l'hôpital, Paris 75013, France; 4 Service d'Anatomopathologie, Groupe Hospitalier Pitié Salpêtrière, AP-HP, GHU EST, 47-83 Boulevard de l'hôpital, Paris 75013, France; 5 Service d'Anatomopathologie, Hôpital Tenon, AP-HP, Cancerest, GHU EST, Université Paris VI, 4 rue de la Chine, Paris 75020, France; 6 Service de Gynécologie Obstétrique, Hôpital Tenon, AP-HP, Cancerest, GHU EST, Université Paris VI, 4 rue de la Chine, Paris 75020, France; 7 Service d'Oncologie Médicale, Hôpital Tenon, AP-HP, Cancerest, GHU EST, Université Paris VI, 4 rue de la Chine, Paris 75020, France

**Keywords:** *MDR1*, *MRP1*, *GSTP1*, rt–PCR, breast cancer, prognosis

## Abstract

Study of the prognostic impact of multidrug resistance gene expression in the management of breast cancer in the context of adjuvant therapy. This study involved 171 patients treated by surgery, adjuvant chemotherapy±radiotherapy±hormonal therapy (mean follow-up: 55 months). We studied the expression of multidrug resistance gene 1 (*MDR1*), multidrug resistance-associated protein (*MRP1*), and glutathione-*S*-transferase P1 (*GSTP1*) using a standardised, semiquantitative rt–PCR method performed on frozen samples of breast cancer tissue. Patients were classified as presenting low or high levels of expression of these three genes. rt-PCR values were correlated with T stage, N stage, Scarff–Bloom–Richardson (SBR) grade, age and hormonal status. The impact of gene expression levels on 5-year disease-free survival (DFS) and overall survival (OS) was studied by univariate and multivariate Cox analysis. No statistically significant correlation was demonstrated between *MDR1*, *MRP1* and *GSTP1* expressions. On univariate analysis, DFS was significantly decreased in a context of low *GSTP1* expression (*P*=0.0005) and high SBR grade (*P*=0.003), size ⩾5 cm (*P*=0.038), high T stage (*P*=0.013), presence of intravascular embolus (*P*=0.034), and >3 N+ (*P*=0.05). On multivariate analysis, *GSTP1* expression and the presence of ER remained independent prognostic factors for DFS. *GSTP1* expression did not affect OS. The levels of *MDR1* and *MRP1* expression had no significant influence on DFS or OS. *GSTP1* expression can be considered to be an independent prognostic factor for DFS in patients receiving adjuvant chemotherapy for breast cancer.

The increasing use of adjuvant chemotherapy in breast cancer, designed to improve both disease-free survival (DFS) and overall survival (OS), highlights the need to develop predictive tests of tumour chemosensitivity in order to identify patients likely or unlikely to benefit from such therapy (Bonadonna *et al*, 1990; Scholl *et al*, 1994). Cytotoxic exposure can induce a multidrug resistance phenotype (MDR), which can involve numerous cell changes (Simon and Schindler, 1994). Some proteins are overexpressed in MDR cell lines, defining a group of MDR-related genes. In humans, *P*-glycoprotein (*P*-gp), encoded by the multidrug resistance gene 1 gene (*MDR1*) (Endicott and Ling, 1989; Gottesman and Pastan, 1993), and multidrug resistance-related protein *MRP1*, first described by Cole *et al* (1994), are two membrane glycoprotein transporters belonging to the more extensive superfamily of ATP-binding cassette (ABC) proteins (Dean *et al*, 2001). More recently, other members of this family have been implicated in multidrug resistance of breast cancer, such as BCRP/ABCG2 and other forms (Leonessa and Clarke, 2003). Such proteins act as energy-dependent efflux pumps capable of expelling a large range of xenobiotics, including doxorubicin and other cytotoxic drugs derived from natural products, out of the cell. MRP1 can act as a transporter of glutathione conjugates (Muller *et al*, 1994). Although the precise role of the glutathione detoxification pathway in the MDR phenomenon has not yet been fully elucidated, the isoenzymes of the glutathione-S-transferase (GSTs), namely the subclass GSTpi (EC 2.5.1.18), have been extensively reported to be overexpressed in tumour cells displaying the MDR phenotype (Keith *et al*, 1990; Buser *et al*, 1997; Ferrandina *et al*, 1997; Frassoldati *et al*, 1997; Silvestrini *et al*, 1997; Boku *et al*, 1998; Stoehlmacher *et al*, 2002; Oudard *et al*, 2002; Cullen *et al*, 2003; Galimberti *et al*, 2003; Bennaceur-Griscelli *et al*, 2004). However, the role of GSTs proteins remains controversial in the literature.

In a previous study, we used a semiquantitative rt–PCR method to evaluate multidrug resistance gene expression in surgical breast cancer biopsies (Lacave *et al*, 1998). The primary objective of the present study was to complete this preliminary study by evaluating the clinical impact of the expression of these genes on the management of breast cancer in a series of 171 patients receiving adjuvant chemotherapy.

## PATIENTS, MATERIALS AND METHODS

### Patients and tissue samples

This study was performed on a series of 171 surgically obtained tumour specimens from 171 patients with stage I–III invasive breast carcinoma, treated between April 1991 and January 2001 by surgery (Surgical Gynecology Departments, at the La Pitié Salpêtrière and Tenon Hospitals), adjuvant chemotherapy (Medical Oncology and Radiation Oncology Department, Tenon Hospital, Paris, France), +/− postoperative radiotherapy (Radiation Oncology Department, Tenon Hospital, Paris, France), +/− hormonal therapy (Medical Oncology and Radiation Oncology Department, Tenon Hospital, Paris, France). The mean and median ages were 54 and 52 years, respectively (range: 34–77), and 54% of patients were postmenopausal. First-line treatment was tumorectomy in 59.6% of patients (*n*=102), and radical mastectomy in 40.4% of patients (*n*=69) with axillary dissection in all cases. All patients received adjuvant chemotherapy. Patients who had received neoadjuvant chemotherapy were excluded from this study. Radiotherapy was performed in 162 cases (94.7%), and 117 patients (68.4%) received hormonal therapy. Details of the systemic treatments used are given in Table 1. Most patients received anthracyclines (*n*=163; 95%). The clinical and pathological characteristics of the study population are also described in Table 1.

Histopathologic typing, Scarff–Bloom–Richardson (SBR) grading and measurement of oestrogen (ER) and progesterone (PR) receptor levels (cutoff value: 10 fmol mg^−1^) were performed by independent investigators. DNA ploidy and S-phase fraction (SPF) were determined by DNA flow cytometry, using a standardised method and consensus rules for interpreting the data (Chassevent *et al*, 2001). Tumours containing a single cell population with a DNA index ranging between 0.9 and 1.05 were classified as diploid, and those with an additional cell population with a DNA index outside the 0.9–1.05 range were defined as aneuploid. The SPF was classified as three SPF classes, defined on the basis of terciles (33rd and 66th percentile) after adjusting for ploidy.

### rt–PCR studies

The conditions and methodological aspects of the end point rt–PCR procedures used in this study have been previously described in detail (Lacave *et al*, 1998). Although real-time PCR has been established as the gold standard rt–PCR procedure for gene expression studies since the end of the 1990s, most patients described here were included in the mid-1990s, at a time when real-time PCR was not routinely performed in the majority of laboratories. We therefore chose to maintain the classical end point rt–PCR method for all of these patients. Briefly, each gene of interest was separately coamplified with its specific endogenous standard (i.e. *MDR1/β*_*2*_*M; MRP1/PGK; GSTπ/PGK*). For *MDR1*, the control cell lines consisted of the drug-sensitive human epidermoid carcinoma KB3-1 cell line and its multidrug-resistant derivative, the KB 8-5 cell line (kindly provided by Dr Gottesman) (Akiyama *et al*, 1985). The MCF7 human breast carcinoma cell line (Soule *et al*, 1973) was obtained from J Robert (Institut Bergonié, Bordeaux, France), and was also used to select the doxorubicin-resistant cell line (MCF7R) used in this study. These cell lines were used as negative and positive controls for GST P1 (*GSTP1*), respectively. The IGROV cell line was obtained from J Bénard (Benard *et al*, 1985), and was used as a negative control *vs* MCF7R to check for *MRP1* expression. Historically, *β*_*2*_*M* has been proposed for use as an internal standard for *MDR1* (Beck *et al*, 1996) when KB cell lines are used as the control cell line (see below). Owing to the lack of *β*_*2*_*M* expression in MCF7R, *PGK* was used as the internal standard for *MRP1* and *GSTP1*. The gene of interest/endogenous standard ratio of test samples was expressed in relation to the ratio found for control cell lines overexpressing the gene tested. Calculated values were expressed in arbitrary units. Control cell lines were also used to determine the standard curves by serial dilutions of total RNA extracted from drug-resistant cell lines, with total RNA extracted from their respective drug-sensitive cell lines in order to validate and standardise the rt–PCR conditions for optimal coamplification of the genes tested and their respective internal control sequences. We had also previously checked that the values obtained for control cell lines always displayed low ranges of interassay variation (coefficient of variation <10% in every case). Owing to the high degree of heterogeneity observed with the rt–PCR procedures used in previous studies to determine the expression of MDR-related genes, no cutoff values have been clearly defined and, in the present study, as shown in Table 2, we chose to define the subgroups on the basis of the estimated median values of gene expression for each gene (⩽ or > the median value) (Table 2).

### Statistical methods

Comparisons between the levels of expression of the three genes studied and patient characteristics were performed using Pearson's *χ*^2^ test. A two-sided *P*<0.05 was considered significant. The DFS was defined as the interval between first treatment and primary failure (local and/or distant recurrence). Actuarial survival rates were computed using the Kaplan–Meier method, and compared using the log rank test (Kaplan and Meier, 1958). The influence of DNA ploidy and SPF fraction on outcome, adjusted for the other prognostic factors, was assessed by univariate and multivariate analysis using the Cox proportional hazards regression model in a forward stepwise procedure (Cox, 1972). The ascending method was used for a block-by-block construction (clinical variables, and then laboratory variables). The various variables were: (a) age at diagnosis (⩽ or >50 years); (b) menopausal status; (c) clinical T stage (T1; T2; T3; T4); (d) histologic type (ductal or lobular carcinoma); (e) histologic lymph node involvement (N−; N+); (f) number of lymph nodes involved (< or ⩾3N+); (g) capsular invasion (CI−; CI+); (h) histologic grade (SBR I–III); (i) surgical margins (⩽ or >1 mm); (j) vascular invasion; (k) associated extensive (>25%) *in situ* carcinoma; (l) intravascular and intralymphatic embolus; (m) hormone receptor status; (n) DNA ploidy; (o) SPF adjusted for ploidy; and expression of (p) *MDR1*; (q) *MRP1* and (r) *GSTP1* genes, each divided into two groups according to whether gene expression was less than or greater than the median value of expression. As most patients received radiotherapy (94.7%) and anthracycline-based adjuvant chemotherapy (95%), we deliberately excluded the type of adjuvant therapy received by the patients from statistical analysis. The study end points compared the levels of expression of each of the three genes with those of the other two multidrug resistance genes, and evaluated the influence of multidrug resistance gene expression on 5-year actuarial DFS, and overall specific survival (OS) rates. Complete information for follow-up and secondary events were obtained for all patients. The median follow-up from the beginning of treatment was 56 months (range: 7–139 months).

## RESULTS

### Tumour characteristics and flow cytometry

Most tumours were ductal (*n*=139; 81%), or lobular carcinomas (*n*=23; 13. 5%). The main characteristics of the samples are summarised in Table 1. Most tumours were early-stage carcinomas (T1–T2: *n*=158; 92%), and were node positive (*n*=106; 62%). The hormonal status was available in 159 patients (93%). In all, 141 samples were evaluated by cytometry. A total of 58 tumours were diploid (41%), and 83 were aneuploid (59%). Only 126 of these 141 tumour samples were available for SPF evaluation, and tumour samples were divided into three SPF prognosis groups: tumours with low SPF values (*n*=35: 28%), intermediate SPF values (*n*=39: 31%) and high SPF values (*n*=52: 41%).

### RT–PCR analysis of tumour samples

Determination of *MDR1* expression was available for 164 tumours (96%). When compared with the negative KB 3.1 and positive KB 8.5 control cell lines, 68 (42%) of tumours did not express the *MDR1* gene, while 96 tumours (58%) expressed *MDR1*. The mean value of the *MDR1*/*β*_*2*_*M* ratio was 0.052±0.008 (range: 0–0.065), with a median of 0.02.

*MRP1* expression was assessed for 131 tumour samples (77%), with a mean *MRP1*/*PGK* ratio of 0.75±0.08 (range: 0–10), and a median of 0.61. Only 10 tumours (7.6%) did not express the *MRP1* gene.

*GSTP1* expression was evaluated in 119 tumour samples (70%), and only three tumours were found not to express this gene. The mean *GSTP1/PGK* ratio was 0.74±0.06 (range: 0–4.6) with a median of 0.63.

Table 3 reports the levels of expression of the three genes in relation to the clinical and laboratory characteristics of patients and samples. No statistically significant difference in the expression of any of the MDR-related genes was observed between any of the subgroups, apart from tumours with negative ER or PR, in which *GSTP1* expression was significantly higher.

When the values were analysed as continuous values, no statistically significant correlation was found between *MDR1*, *MRP1* and *GSTP1* expression.

### Patient outcome

Nine (5.5%) patients developed local recurrence after a mean interval of 27.5 months (range: 2–49 months), three (1.7%) patients developed a regional axillary relapse (mean interval: 29 months, range: 9–53 months), and 24 patients (14%) developed distant metastasis after a mean interval of 36 months (range: 3–83 months). In all, 18 patients had died at the endpoint date of this analysis: 17 from cancer (10%) and one from another cause. A total of 16 (9.3%) patients developed a second cancer (breast and/or another primary tumour).

The 5-year DFS rate was 79.7% (±3.3; [73.3–86.6]) in the overall population, 82% (±5.6; [71.7; 93.7]) among node-negative patients, and 79.3% (±4.2; [71.5–87.9]) among node-positive patients (*P*=0.38). The 5-year specific survival was 89.7% (±2.7; [84.5–95.2]) in the overall population, 93.3% (±3.9; [85.9–1]) among node-negative patients, and 88.3% (±3.6; [81.6–95.6]) among node-positive patients (*P*=0.26).

### Prognostic impact of multidrug resistance genes

**Figure 1:**
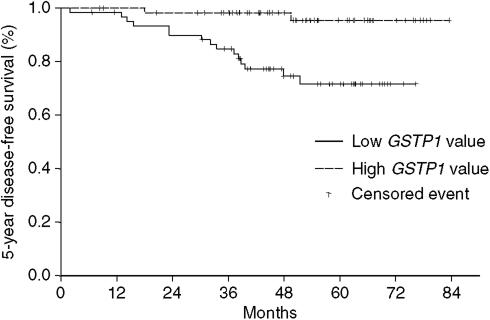
5-year disease-free survival according to level of *GSTP1* expression.

In the overall population, univariate Cox analysis showed a better 5-year DFS rate in the group of patients with high *GSTP1* expression than in the group with low *GSTP1* expression (95.4±0.03% [89.2–100] versus 71.9±0.06% [60.4–85.6]; HR=0.33; *P*=0.0005, Figure 1). Other factors found to be significantly correlated with DFS on univariate analysis were SBR grade (*P*=0.039), T stage (*P*=0.013), more than three positive nodes (*P*=0.05), and presence of intravascular and intralymphatic embolus (*P*=0.034) (Table 4). Expression of the other two multidrug resistance genes, *MDR1* and *MRP1*, did not influence 5-year DFS.

On multivariate analysis (Table 5), complete clinical and laboratory data were available for 90 patients. In the overall population, ER receptor status and subgroups based on *GSTP1* expression were shown to be independent predictors for DFS (*P*=0.002 and 0.011, respectively).

On univariate analysis, only two factors statistically influenced the 5-year OS rate: SBR grade (*P*=0.028), and clinical lymph node status (N0 *vs* N1, *P*=0.039). Expression of the three multidrug resistance genes studied did not influence the 5-year OS. Cox multivariate analysis did not demonstrate any factor independently correlated with 5-year OS.

## DISCUSSION

The increasing use of chemotherapy in breast cancer has led physicians to develop accurate and reliable tests to identify MDR determinants in clinical studies. However, very limited data are available in the literature in this field and the precise mechanism of action, the relationship between multidrug resistance genes, and their clinical impact on the outcome of patients with breast cancer remain unclear, as published results have been discordant (Keith *et al*, 1990; Buser *et al*, 1997; Frassoldati *et al*, 1997; Silvestrini *et al*, 1997; Trock *et al*, 1997; Ferrero *et al*, 2000; Leonessa and Clarke, 2003). Few data are available concerning the impact of multidrug resistance genes on the clinical outcome of patients treated for breast cancer. This study was mainly designed to evaluate the clinical impact (DFS and OS) of *MDR1*, *MRP1* and *GSTP1* gene expression on the management of patients with breast cancer treated by adjuvant chemotherapy.

When the values were considered as continuous values, correlation studies did not reveal any statistically significant correlation between *MDR1*, *MRP1*, and *GSTP1* expressions. In the previous study, published in 1998, based on a series of 74 patients, we observed a significant positive correlation between *MRP1* and *GSTpi* expression (Lacave *et al*, 1998). This correlation was not confirmed in the present study, possibly because of a more scattered distribution of expression levels. Although GSH has been demonstrated to be necessary for MRP1-mediated cellular efflux of certain natural substances (Rappa *et al*, 1997) and although *in vitro* detoxification of anticancer agents involves a combined action of GSTs and MRPs (Morrow *et al*, 1998; O'Brien *et al*, 2000; Depeille *et al*, 2004; Depeille *et al*, 2005), a clear-cut correlation between *MRP1* and *GSTP1* expression has yet to be established in the clinical setting.

In this series, the 5-year DFS and OS were not influenced by the expression of either *MDR1* or *MRP1.* In a series of 85 node-positive breast cancer patients receiving anthracycline-based adjuvant therapy, Ferrero *et al* (2000) did not find any significant influence of *MDR1* and *MRP1* on progression-free or overall specific survival, and Kanzaki *et al* (2001) did not observe any correlation between *MRP1* mRNA expression and relapse after doxorubicin adjuvant therapy. In contrast, in a series of 59 breast cancer patients, Burger *et al* (2003) reported a clear link between RNA expression of lung resistance-related protein and *MDR1*, and progression-free survival, but this series included only advanced cases. In a recent publication based on 104 patients treated for breast cancer, higher MDR1/P-gp expression was associated with a statistically significant shorter OS and progression-free time, but the authors used a method based on immunohistochemical reactions using monoclonal antibodies (Surowiak *et al*, 2005). In a study of 27 patients, the risk of relapse in the 10 years following adjuvant chemotherapy was higher in patients whose primary tumours expressed higher levels of *MRP1* mRNA (Ito *et al*, 1998). Heterogeneous results are a common feature of studies evaluating the expression and prognostic role of this gene, mainly due to both methodological and biological factors, and the prognostic impact of these two genes on DFS or OS remains to be established (Leonessa and Clarke, 2003).

The main result emerging from this study is that *GSTP1* expression can be taken into account in the management of breast cancer patients receiving adjuvant chemotherapy.

In this study, we clearly demonstrated, on both univariate and multivariate Cox analysis, that subgroups based on *GSTP1* expression were shown to be independent predictors of DFS, with a better 5-year DFS rate in the group of patients with high *GSTP1* expression than in the group with low *GSTP1* expression. This finding supports the results of previous studies concerning various type of tumours (Goasguen *et al*, 1996; Buser *et al*, 1997; Howells *et al*, 1998; Kearns *et al*, 2001). For example, Buser *et al* (1997), in a series of 89 women with untreated breast cancer, found that high levels of *Gst* and *Gpx* activities were associated with favourable clinical characteristics and a good prognosis, whereas low levels of *Gst* activity were associated with more aggressive or more advanced disease, although the results did not reach the limit of statistical significance. However, the results of our study are somewhat different from those reported by Buser *et al*. In our study, all patients had received adjuvant chemotherapy, whereas in Buser's study, only a small minority of patients had received adjuvant chemotherapy. The mean age of the population was also considerably higher in the study by Buser *et al*. This might explain why *GSTP1* was found to be a significant prognostic factor on both univariate and multivariate analysis.

The precise molecular mechanism responsible for this phenomenon is unclear. As *GSTP1* is the major GSTs consistently expressed in both normal and tumour breast tissue (Forester, *Carcinogenesis* 1990; **11**: 2163–2170), it can be hypothesised that low *GSTP1* expression would reduce the global activity of GSTs, and consequently reduce glutathione (GSH) consumption in GST-catalysed reactions, thereby leading to higher levels of GSH, which would block apoptosis and promote proliferation of tumour cells. This hypothesis was first proposed by Kearns *et al* (2001), who reported an association between elevated GSH levels in leukaemia cells and an increased risk of relapse in childhood acute lymphoid leukaemia. Morales *et al* (2005) recently confirmed these results, by showing that intracellular glutathione levels determine cell sensitivity to drug-induced apoptosis. It has also been demonstrated that intracellular GSH depletion of human oral squamous cell carcinoma by inorganic selenium compounds may cause caspase-9-mediated apoptosis (Takahashi *et al*, 2005).

A possible explanation for the increased DFS in patients with high levels of *GSTP1* expression could involve the role of GSTP1 on cell proliferation. GSTP1 has been identified as a modulator of cell signaling, by interacting with and inhibiting c-Jun N terminal kinase (JNK) (Adler *et al*, 1999; Elsby *et al*, 2003) implicated in the control of cell proliferation (Timokhina *et al*, 1998; Tournier *et al*, 2000) and transformation (Raitano *et al*, 1995; Xiao and Lang, 2000). These results are consistent with the work by Ruscoe *et al* (2001), showing that GST-depleted cells, which exhibited a higher JNK activity, proliferated faster than their wild-type counterparts.

An hypothesis can be proposed to explain the molecular mechanisms responsible for higher levels of expression of *GSTP1* in patients with better DFS, as the very variable levels of expression of *GSTP1* cannot be explained by the presence of variant genotypes previously implicated in the pathogenesis of breast cancer in patients treated with chemotherapy (Yang *et al*, *Cancer* 2005; **103**: 52–58), as these variants are mainly related to variations of enzyme catalytic activity (Sweeney, *Cancer Res* 2000; **60**: 5621–5624). Complementary studies are currently underway to test this hypothesis, which was not included in the initial design of our study. Altered DNA methylation, related to the fundamental role of epigenetic events in cancer (Jones and Baylin, 2002; Verma *et al*, 2004) now constitutes a growing field of clinical investigation in cancer (Das and Singal, 2004) and could possibly explain variable levels of *GSTP1* expression in our patients. Altered DNA methylation has been well documented in breast cancer (for review see Widschwendter and Jones, 2002). In particular, inactivation of *GSTP1* by promoter hypermethylation was initially reported to be frequent in renal carcinoma and in about 30% of primary breast cancers by Esteller *et al* (1998). Hypermethylation of CpG dinucleotides at the 5′ transcriptional regulatory region has been shown to be sufficient to inhibit *GSTP1* transcription in the MCF-7 breast cancer cell line when mediated by the methyl-CpG-binding domain (MBD) protein MBD2 (Lin and Nelson, 2003). Jhaveri and Morrow (1998) previously showed that the *GSTP1* CpG island is hypermethylated in ER-positive, *GSTP1*-nonexpressing MCF-7, but is undermethylated in ER-negative, *GSTP1-*expressing cell lines. Note that, in our study, both ER- and PR-negative patients exhibited higher levels of *GSTP1* expression. In a recent study, Shinozaki showed that hypermethylation of *GSTP1* was significantly associated with macroscopic sentinel lymph node metastasis compared to patients with microscopic or no sentinel lymph node (Shinozaki *et al*, 2005).

Most authors have studied the relevance of MDR phenotype as a predictive test for breast cancer response in patients treated by neoadjuvant chemotherapy, and have found that MDR phenotype is indeed a relevant tool for monitoring breast cancer response to this treatment (Chevillard *et al*, 1996; Trock *et al*, 1997; Burger *et al*, 2003). In this study, we deliberately excluded patients who had received neoadjuvant chemotherapy in order to obtain a homogeneous population. This subject will be further developed in a study to be published subsequently.

In conclusion, our findings suggest that a low level of *GSTP1* gene expression is an independent predictive factor of poor 5-year DFS in patients treated by adjuvant chemotherapy for breast cancer. *MDR1*, *MRP1* did not show any significant influence on the prognosis of these patients.

## Figures and Tables

**Table 1:** Patient characteristics

	**Patients (*n*)**		**Patients (*n*)**
*Age (year)*		*SBR grade*	
⩽55	102	I	28
>55	69	II	73
		III	67
		Not available	3
			
*Postmenopausal*		*DNA ploidy*	
Yes	92	Diploid	58
No	79	Aneuploid	83
		Not available	30

*Clinical T stage*		*S-phase fraction*	
T1	85	Low	35
T2	73	Medium	39
T3	6	High	52
T4	7	Not available	45

*Histological nodal status*		*Oestrogen receptors*	
N−	64	Yes	111
N+	106	No	48
Not available	1	Not available	12

*Number of positive nodes*		*Progesterone receptors*	
<3 N+	66	Yes	87
⩾3 N+	39	No	68
		Not available	16

*Capsular invasion*		*Postoperative radiotherapy*	
C I−	116	Yes	162
C I+	54	No	9
Not available	1		

*Histologic type*		*Adjuvant hormonal therapy*	
Ductal	139	Yes	117
Lobular	23	No	51
Others	9	Not available	3
			
*Associated extensive in situ carcinomas*		*Type of chemotherapy*	
Yes	44	FEC	147
No	123	THEP VCF	15
Not available	4	FNC	7
		EP	1
		CMF	1

*Intravascular embolus*			
Yes	49		
No	107		
Not available	15		

FEC=fluorouracil, epirubicin, cyclophosphamide; THEPVCF=theprubicin, vincristine, cyclophosphamide, fluorouracil; CMF=cyclophosphamide, methotrexate, fluorouracil; FNC=fluorouracil, novantron, cyclophosphamide; EP=epirubicin, paclitaxel.

**Table 2:** Definition of subgroups according to the median value of gene expression

	**Gene expression**	
	**Low**	**High**
*MDR1*	⩽2% (*n*=93)	>2% (*n*=72)
*MRP1*	⩽61.7% (*n*=105)	>61.7% (*n*=66)
*GSTP1*	⩽63% (*n*=61)	>63% (*n*=59)

**Table 3:** MDR phenotype according to patient and tumor characteristics

	**MDR1**		**MRP**		**GSTp**	
*Age (year)* a						
⩽55	5.86±1.12	0.288	83.79±7.77	0.745	70.3±16.91	0.459
>55	4.18±0.95		63.82±6.38		80.1±11.22	
						
*Postmenopausal*						
Yes	3.97±0.75	0.09	70.96±7.85	0.976	81.3±9.44	0.222
No	6.59±1.42		81.78±18.15		65.18±8.44	
						
*Clinical T stage*						
T1	4.95±1.04	0.534	104.21±17.93	0.029	77.1±9.92	0.362
T2	4.83±1.09		54.74±6.1		69.51±7.19	
T3	11.2±9.49		35.53±15.57		123.8±84.76	
T4	7.25±5.1		43.5±18.28		49.5±20.41	
						
*Histologic type* b						
Ductal carcinomas	5.61±0.93	0.289	81.82±10.68	0.284	73.04±6.9	0.524
Lobular carcinomas	3.32±1.38		53.08±11.93		85.31±22.6	
						
*SBR grade* c						
SBRI	5.13±2.16	0.804	71.83±13.7	0.933	89.63±21.75	0.622
SBRII	4.74±0.96		82.37±19.41		66.4±8.86	
SBRIII	5.87±1.42		70.02±7.82		76.83±9.31	
						
*Histologic nodal status*						
N−	6.18±1.58	0.326	79.67±19.59	0.465	85.15±10.0	0.230
N+	4.59±0.8		72.04±7.54		68.91±8.56	
						
*Intravascular embolism*						
Yes	5.28±1.39	0.684	73.66±11.83	0.963	57.07±10.68	0.08
No	4.63±0.87		75.16±12.53		82.7±8.4	
						
*Associated extensive in situ carcinoma*						
Yes	6.04±1.58	0.56	77.04±12.9	0.56	71.32±13.25	0.7
No	4.98±0.91		74.84±11.31		77.33±7.71	
						
*Oestrogen receptor status*						
Negative	7.45±2.04	0.08	60.67±5.16		99.09±12.3	
Positive	4.29±0.78		79.31±12.56	0.59	65.39±8.05	0.028
						
*Progesterone receptor status*						
Negative	5.51±1.52	0.835	67.99±8.29	0.571	87.04±8.7	0.038
Positive	5.15±0.95		79.43±16.72		59.99±9.47	
						
*DNA ploidy*						
Diploid	5.45±1.57	0.735	69.63±10.44	0.164	68.19±11.26	0.298
Aneuploid	6.10±1.15		61.51±5.85		84.31±9.84	
						
*S-phase fraction*						
Low	5.1±1.43	0.312	75.66±11.11	0.580	84.32±15.63	0.719
Medium	4.52±1.59		71.79±13.61		81.72±13.68	
High	7.95±1.96		61.29±6.4		70.8±7.4	

a Patient's age.

b Other histologic types were excluded.

c SBR: Scarff–Bloom–Richardson index.

Values are expressed as mean±s.e. of RNA levels of *MDR1, MRP1* and *GSTP1* rt–PCR products, expressed as a percentage of control cell lines. One-way analysis of variance was used to compare these variables.

**Table 4:** 5-year disease-free survival rates and Cox univariate analysis

	***n* pts**	**5-year DFS (±s.e.) (%) [CI]**	***P*; HR**
*GSTπ*			0.0005; 0.33
Low expression	60	71.9 (±6.3) [60.4–85.6]	
High expression	60	95.4 (±3.3) [89.2–100]	
NA	51		
			
*T Stage*			0.013; 2.48
T1	85	87.4 (±3.7) [80.4–95.1]	
T2	73	77 (±6) [66.1–89.8]	
T3	6	25.5 (±26.3) [4–100]	
T4	7	46.8 (±18.2) [21.9–100]	
			
*Intravascular embolus*			0.034; 1.53
Yes	49	70.4 (±7) [57.9–85.7]	
No	107	86.9 (±3.7) [80.1–94.2]	
			
*SBR grade*			0.039; 1.75
SBRI	28	100	
SBRII	73	74.6 (±5.6) [64.4–86.5]	
SBRIII	67	77.2 (±5.4) [67.2–88.7]	
			
>*3 N*+			0.05; 2.17
Yes	39	71.3 (±7.3) [58.2–87.2]	
No	66	83 (±3.7) [75.9–90.7]	

NA=not available; N Pts=number of patients; SBR=Scarff–Bloom–Richardson; T=T stage according to TNM AJCC 2002 classification; [CI]=confidence interval; HR=hazard ratio.

>3N+=more than three positive nodes involved.

**Table 5:** Cox multivariate analysis for 5-year disease-free survival

	**Overall population (*n*=90)**	
	**Relative risk (s.e.)**	***P***
Oestrogen receptor status	0.066 (0.876)	0.002
*GST P1* level expression (⩽ *vs* >63%)	0.302 (0.469)	0.011

Model for overall population included the following factors (factors with a *P*-value <0.2 using univariate analysis): SBR grade, T stage, clinical and histologic node involvement, number of lymph nodes involved (< or ⩾3 N+), capsular invasion, presence of intravascular or intratumoral embolus, presence of intraductal or intralobular extensive carcinoma, oestrogen receptor status, *MDR1* and *GSTP1* gene expression.
